# A Novel lncRNA Panel Related to Ferroptosis, Tumor Progression, and Microenvironment is a Robust Prognostic Indicator for Glioma Patients

**DOI:** 10.3389/fcell.2021.788451

**Published:** 2021-12-07

**Authors:** Yikang He, Yangfan Ye, Wei Tian, Huaide Qiu

**Affiliations:** ^1^ Department of Rehabilitation, Zhongda Hospital, School of Medicine, Southeast University, Nanjing, China; ^2^ Department of Neurosurgery, The First Affiliated Hospital of Nanjing Medical University, Nanjing, China; ^3^ The First Clinical Medical College, Nanjing Medical University, Nanjing, China; ^4^ Center of Rehabilitation Medicine, The First Affiliated Hospital of Nanjing Medical University, Nanjing, China

**Keywords:** long non-coding RNAs, prognostic signature, glioma, the cancer genome atlas, chinese glioma genome atlas

## Abstract

**Objective:** To establish a lncRNA panel related to ferroptosis, tumor progression, and microenvironment for prognostic estimation in patients with glioma.

**Methods:** LncRNAs associated with tumor progression and microenvironment were screened via the weighted gene co-expression network analysis (WGCNA). Overlapped lncRNAs highlighted in WGCNA, related to ferroptosis, and incorporated in Chinese Glioma Genome Atlas (CGGA) were identified as hub lncRNAs. With expression profiles of the hub lncRNA, we conducted the least absolute shrinkage and selection operator (LASSO) regression and built a ferroptosis-related lncRNA signature to separate glioma patients with distinct survival outcomes. The lncRNA signature was validated in TCGA, the CGGA_693, and CGGA_325 cohorts using Kaplan-Meier survival analysis and ROC curves. The ferroptosis-related lncRNA panel was validated with 15 glioma samples using quantitative real-time PCR (qRT-PCR). Multivariate Cox regression was performed, and a nomogram was mapped and validated. Immune infiltration correlated to the signature was explored using TIMER and CIBERSORT algorithms.

**Results:** The present study identified 30 hub lncRNAs related to ferroptosis, tumor progression, and microenvironment. With the 30 hub lncRNAs, we developed a lncRNA signature with distinct stratification of survival chance in patients with glioma in two independent cohorts (HRs>1, *p* < 0.05). The lncRNA signature revealed a panel of 14 lncRNAs, i.e., APCDD1L-AS1, H19, LINC00205, LINC00346, LINC00475, LINC00484, LINC00601, LINC00664, LINC00886, LUCAT1, MIR155HG, NEAT1, PVT1, and SNHG18. These lncRNA expressions were validated in clinical specimens using qRT-PCR. Robust predictive accuracies of the signature were present across different datasets at multiple timepoints. With univariate and multivariate regressions, we demonstrated that the risk score based on the lncRNA signature is an independent prognostic indicator after clinical factors were adjusted. A nomogram was constructed with these prognostic factors, and it has demonstrated decent classification and accuracy. Additionally, the signature-based classification was observed to be correlated with multiple clinical characteristics and molecular subtypes. Further, extensive immune cells were upregulated in the high-risk group, such as CD8^+^ T cell, neutrophil, macrophage, and myeloid dendritic cell, indicating increased immune infiltrations.

**Conclusion:** We established a novel ferroptosis-related lncRNA signature that could effectively stratify the prognosis of glioma patients with adequate predictive performance.

## Introduction

Glioma is the most common intracranial malignancy, accounting for 80% of brain malignancies and 30% of brain tumors (de Robles et al., 2015). Adult diffused gliomas were classified into lower-grade glioma (LGG) and glioblastoma multiforme (GBM), as indicated in the 2016 World Health Organization (WHO) classification (Louis et al., 2016; Wen and Huse, 2017). While GBM presents an inferior prognosis with a median survival of 14.6 months (Lara-Velazquez et al., 2017), most LGG progresses to GBM, leading to an unfavorable overall survival rate (Ostrom et al., 2014; Claus et al., 2015; Lim et al., 2018; Kirby and Finnerty, 2020). Interestingly, patients with LGG could decease within months post-surgery while those with GBM lived for years in some cases, which indicates that the current classification of gliomas does not reflect the distinct survival outcomes (Chen et al., 2017). To evaluate prognosis and develop therapeutic strategies, molecular biomarkers including O6-methylguanine-DNA methyltransferase (MGMT) methylation, codeletion of chromosome 1 and 19 (1p/19q), mutations in isocitrate dehydrogenase (IDH) are under active investigation in patients with glioma (Molinaro et al., 2019). Although these molecular biomarkers are implicated in the regulation of cancer cell proliferation and death (Ceccarelli et al., 2016), it remains a challenge to inform individual prognosis and treatment. Therefore, novel biomarkers are warranted for prognostic stratification and therapeutic strategies.

Ferroptosis, a novel form of programmed cell death, is characterized by the aberrant accumulation of lipid peroxide related to iron metabolism (Dixon et al., 2012). Various types of cancer cells were observed to be sensitive to ferroptosis (Eling et al., 2015; Belavgeni et al., 2019; Carbone and Melino, 2019), which may alter the process of cancer development and therapies (Gan, 2019; Liang et al., 2019; Stockwell and Jiang, 2019). Additionally, genes with regulatory effects on ferroptosis, such as P53 (Junttila and Evan, 2009), DPP4 (Enz et al., 2019), and HSPB1 (Arrigo and Gibert, 2012), are associated with the development and progression of cancers. Besides, ferroptosis-related genes have shown potentials to be prognostic biomarkers in glioma (Liu et al., 2020; Zhuo et al., 2020), indicating the significance of ferroptosis in glioma.

Long non-coding RNAs (lncRNAs) are a type of transcripts with more than 200 nucleotides which does not code for proteins (Clark et al., 2012). lncRNAs are the major component of human transcriptome with a proportion up to 80% (Hong et al., 2020). Owing to the ubiquitous regulatory effect of lncRNAs, it is involved in tumorigenesis in numerous cancers. Moreover, dysregulation of lncRNAs has been reported to be associated with the pathogenicity of gliomas (Bhan et al., 2017). Further, there has been emerging reports of lncRNA signatures in predicting survival of glioma patients (Wang et al., 2018; Luan et al., 2019; Xia et al., 2020). A ferroptosis-related lncRNAs signature was observed to be associated with prognosis, tumor microenvironment, and response to radiotherapy in glioma (Zheng et al., 2021). However, the previous study used median risk scores for each dataset, leading to half-by-half classifications and potential false-positive results. In machine learning models, a consistent cutoff value for different datasets enhances generalizability, and thereby increasing applicability in real world.

In the present study, we sought to establish a ferroptosis-related lncRNA signature with transcriptomic and clinical data from the Cancer Genome Atlas (TCGA) and two cohorts from the Chinese Glioma Genome Atlas (CGGA). We analyzed the expression profiles of ferroptosis-related lncRNAs and fitted key lncRNAs into a prediction model, which was rigorously validated in three datasets with the same cutoff value. Our data indicate that the ferroptosis-related lncRNA signature is ready to serve as a novel prognostic panel, and therapies targeting these lncRNAs might yield effective results.

## Materials and Methods

### Data Collection

RNA sequencing (RNA-seq) data and corresponding clinical information of 698 glioma samples were taken from TCGA (https://portal.gdc.cancer.gov), while the test datasets with 693 glioma samples (CGGA_693) as well as with 325 samples (CGGA_325) was retrieved from the CGGA (http://www.cgga.org.cn). Then ferroptosis-related genes were retrieved from previous literature (Liu et al., 2020).

### Coexpression Network Identified Clinically Relevant Long Non-Coding RNAs

The lncRNAs associated with tumor progression and microenvironment were screened using the weighted gene co-expression network analysis (WGCNA) package (Langfelder and Horvath, 2008). Samples and lncRNAs were filtered with statistical tests, while outliers were detected with hierarchical clustering. Then we selected the soft threshold and engendered the hierarchical clustering tree. Module eigengene (ME) representing the overall expression level of the module was calculated, and the Pearson correlation coefficients between lncRNA modules and clinical traits were calculated to identify modules of interest. Modules with correlation coefficients >0.5 with regarding to tumor grade and ESTIMATE scores were identified, and lncRNAs in these modules were identified as clinically relevant lncRNAs.

Subsequently, we calculated the correlation coefficients between each gene expression in the specific module and clinical trait, and the value of coefficients were defined as gene significance (GS). Module memberships (MMs) were defined as the coefficients between gene expression and MEs. Correlations between GS and MMs were analyzed to verify the significance of lncRNAs in the specific module.

### Identification of Hub Long Non-Coding RNAs and Development of a Prognostic Signature

Based on the list of clinically relevant lncRNAs, we identified specific ferroptosis-related lncRNAs whose expression was correlated to ferroptosis-related genes (Pearson Cor>0.5 and *p* < 0.05). Overlapped lncRNAs indexed in the ferroptosis-related lncRNA list, highlighted in WGCNA, and incorporated in CGGA were identified as hub lncRNAs. With expression profiles of the hub lncRNA, we conducted the least absolute shrinkage and selection operator (LASSO) regression analysis (Meier et al., 2008) and selected key lncRNAs in TCGA dataset. Subsequently, we calculated the individualized risk score with coefficients and expression levels of the key lncRNAs, which stands for a ferroptosis-related prognostic signature that separates the high-risk glioma patients from the low-risk ones. The formulae for risk score were as follows.

$$\text{risk}\;{\text{score}=\text{Σβ}}_{\text{i}}{\text{*X}}_{\text{i}}$$

(X_i_ represents the identified hub lncRNA expression, and β_i_ represents the corresponding coefficient.)

TCGA-glioma patients were divided into high-risk and low-risk groups based on the median risk score as the cutoff. Then the clinical relevance was validated using Kaplan-Meier survival analysis between risk groups. The prognostic signature was presented as a risk plot that includes the distribution of risk score and classification of survival status for patients in different groups. The time-dependent receiver operating characteristic (ROC) analysis was performed (Heagerty et al., 2000), and the area under the curve (AUC) was calculated to evaluate the prognostic accuracy in the TCGA-glioma patients at 1-year, 3-years, and 5-years post-operation. AUC>0.7 was considered adequate accuracy. The prognostic signature with the same risk score formula and cutoff value was then validated in the CGGA-glioma patients. It was then validated in CGGA using Kaplan-Meier survival analysis, ROC curves, as well as a risk plot.

### The Independently Prognostic Value of the Signature

The univariate and multivariate Cox regressions were conducted to determine whether the prognostic signature was independent of clinical characteristics as well as to identify other independent prognostic factors. To determine whether the signature was independent of additional treatment, we also conducted subgroup survival analysis with variables of chemotherapy and radiotherapy. A nomogram was constructed with these prognostic factors using the rms R package, and it was validated using calibration plot (Qiu et al., 2020). Additionally, the correlation between the prognostic signature and clinical characteristics were explored by chi-square test using TCGA.

### Quantitative Real-Time PCR Analysis of Long Non-Coding RNAs in Glioma Tissues

A total of 15 glioma specimens (nine LGG vs six high-grade gliomas [HGG]) were obtained from the Department of Neurosurgery, First Affiliated Hospital of Nanjing Medical University. All specimens were stored in liquid nitrogen, and all patients provided informed consent. The collecting of specimens was approved by the Ethics Committee of First Affiliated Hospital of Nanjing Medical University (No: 2020-SRFA-167).

Total RNA extraction was performed by standard procedures. quantitative real-time PCR (qRT-PCR) was conducted using Oligo primers (Tsingke Biotechnology, for indicated lncRNAs with AceQ qPCR SYBR Master Mix (Vazyme, Q141-02). In all assays, GAPDH served as normalization control. Data were analyzed using the −ΔΔCt method with each test performed in triplicate.

### Immune Infiltration Correlated to the Long Non-Coding RNAs Signature

To explore the immune infiltration in the glioma samples stratified by the signature, immune cell abundances were calculated using TIMER (Li et al., 2020) and CIBERSORT (Newman et al., 2015). Between-group comparisons were conducted using Wilcoxon test, and immune cell types with *p* < 0.05 were kept for visualization.

### Statistical Analysis

Cox regression analysis and Kaplan-Meier method were performed for survival analysis, where Cox *p*-values and log-rank *p*-values were calculated. Pearson correlation was applied to determine significant correlations between continuous variables, while chi-square test was adopted to delineate correlations between categorical variables. PCR data were analyzed with independent *T* tests between groups. Multiple *T* tests were corrected using the Two-stage linear step-up procedure of Benjamini, Krieger and Yekutieli, with *q* = 1% being statistically significant (Benjamini et al., 2006). Data were analyzed using R software 4.1.1 and GraphPad Prism 8.3.0, where *p*-value<0.05 was regarded as statistically significant.

## Results

### Identification of Hub Long Non-Coding RNAs and Enrichment Analysis

**FIGURE 1:**
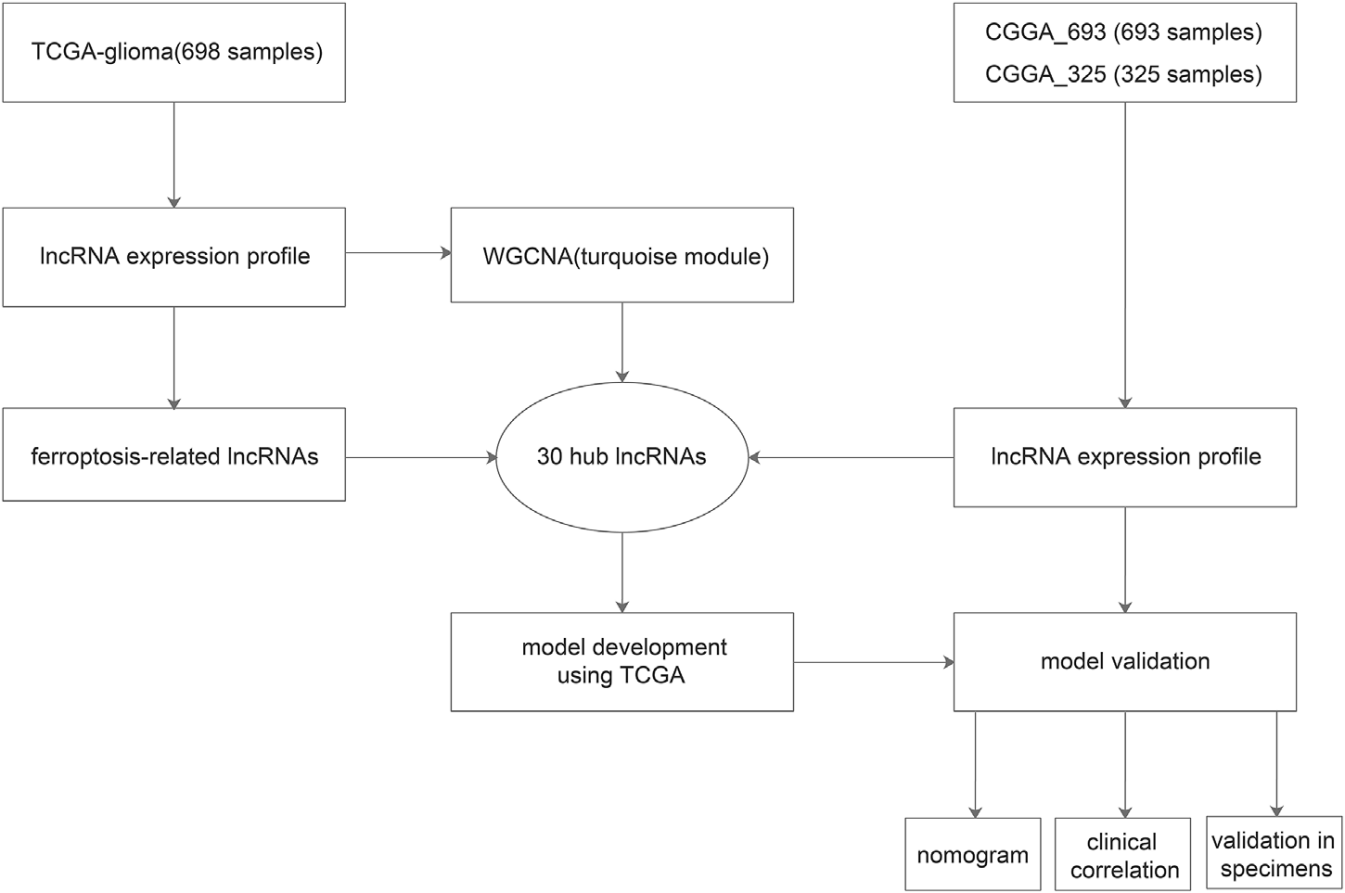
Work flow of the present study.

**FIGURE 2:**
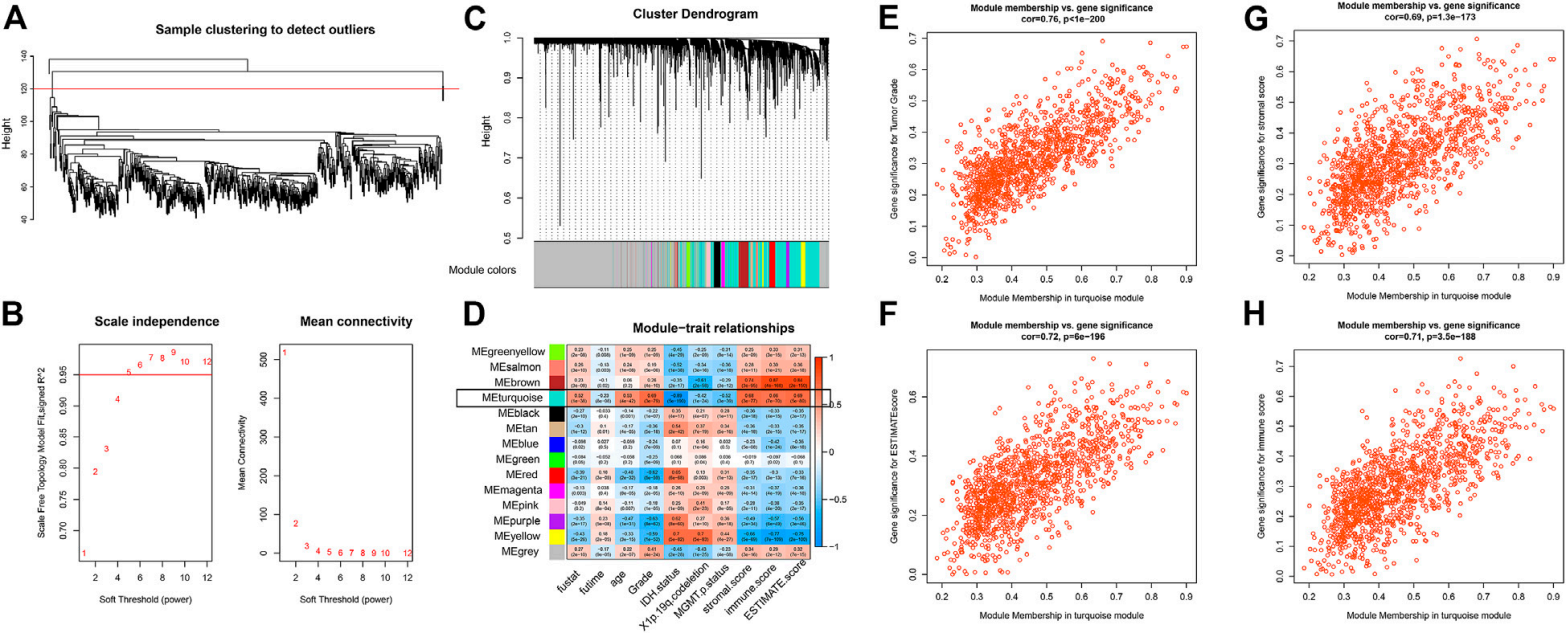
WGCNA using lncRNA expressions **(A)** Sample clustering to detect outliers **(B)** Soft threshold selection **(C)** Cluster dendrogram to distinguish modules **(D)** Correlation matrix between modules and clinical traits **(E–H)** Scatter plot with GS and MM in the turquoise module with regard to tumor grade **(E)**, ESTIMATE combined score **(F)**, ESTIMATE stromal score **(G)**, and ESTIMATE immune score **(H)**.

The flow chart of the present study is presented in Figure 1. Using the WGCNA package, we performed the hierarchical clustering and detected one outlier (Figure 2A). With the cutoff value of 0.95 in Scale-free *R* ^2^, the soft-thresholding power was set to be five (Figure 2B). The clustering dendrograms identified 14 modules (Figure 2C), out of which the turquoise module was significantly correlated with tumor grade as well as tumor microenvironment (Cor>0.5, Figure 2D). To be specific, features including tumor grade, ESTIMATE combined score, stromal score, and immune score were highlighted. Scatterplots of GS for the aforementioned features versus MM in the turquoise module exhibit a marked correlation, suggesting the functional significance of these lncRNAs in the turquoise module (Figures 2E–H). Therefore, a total of 1223 clinically relevant lncRNAs embedded in the turquoise module were selected.

### Development and Validation of the Prognostic Signature

**FIGURE 3:**
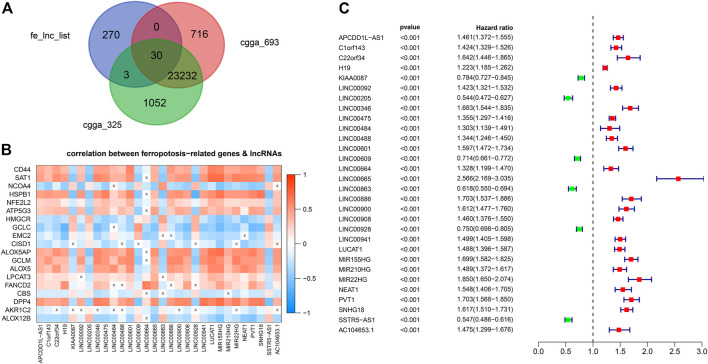
Identification of hub lncRNAs **(A)** Venn Gram for identification of hub lncRNAs **(B)** Correlation matrix of hub lncRNAs and genes implicated in ferroptosis **(C)** Forest plot showing prognostic value of the 30 hub lncRNAs.

**FIGURE 4:**
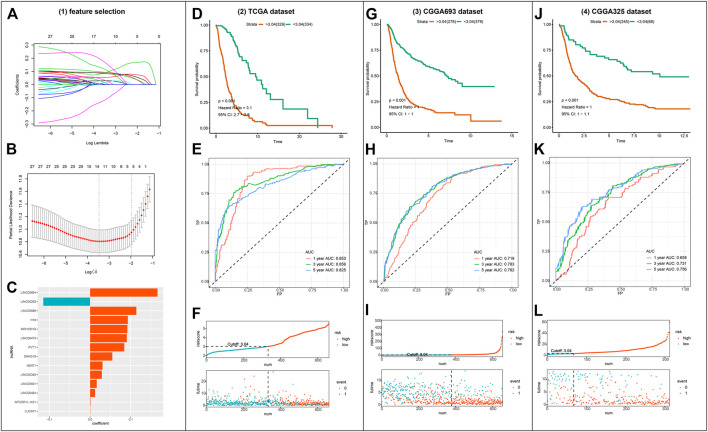
Construction and validation of the ferroptosis-related lncRNA signature **(A–B)** Visualization of LASSO regression **(C)** Coefficients of the 14 lncRNAs fitted in the signature **(D)** Kaplan-Meier curve presenting survival of high-risk and low-risk groups in TCGA **(E)** Time-dependent ROC for the signature in TCGA **(F)** Discriminative plot of the signature using TCGA **(G, J)** Kaplan-Meier curve presenting survival of high-risk and low-risk groups in CGGA_693 or CGGA_325 respectively **(H, K)** Time-dependent ROC for the signature in CGGA_693 or CGGA_325 respectively **(I, L)** Discriminative plot of the signature using CGGA_693 or CGGA_325 separately.

Out of the 1223 lncRNAs in the turquoise module, 303 lncRNAs were related to ferroptosis. After intersecting the 303 ferroptosis-related lncRNAs with the gene list of CGGA, we retrieved 30 hub lncRNAs (Figure 3A). The correlation between the expressions of 30 hub lncRNAs (columns) and ferroptosis-related genes (rows) was presented in Figure 3B. The forest plot showing prognostic value of the 30 hub lncRNAs was presented in Figure 3C. With the 30 hub lncRNAs, we selected 14 key lncRNAs with Lasso regression using TCGA dataset (Figures 4A,B). As shown in Figure 4C, the 14 lncRNAs were as follows (with absolute value of model coefficients increasing): APCDD1L-AS1, H19, LINC00205, LINC00346, LINC00475, LINC00484, LINC00601, LINC00664, LINC00886, LUCAT1, MIR155HG, NEAT1, PVT1, and SNHG18. Except for LINC00205 exhibits a negative coefficient on risk score, which indicates its protective effects on prognosis, the other lncRNAs were observed to be risk factors with positive coefficients (Figure 4C). Subsequently, we calculated individualized risk scores with coefficient-weighted expression levels of 14 lncRNAs after extracting the coefficients. TCGA-glioma patients were divided into high-score and low-score groups based on the median risk score of 3.04 as the cutoff. Significant difference was shown between these two groups in survival analysis (Figure 4D, HR = 3.1, 95% CI = 2.7–3.6, *p* < 0.001). ROC curve analysis of the signature in TCGA-glioma patients achieved AUC of 0.853, 0.856, and 0.825 at 1-, 3- and 5-years (Figure 4E). The risk plot presented a clear separation of survival status between risk groups with red dots being ceased cases and blue ones alive (Figure 4F). Likewise, we calculated individualized risk scores in CCGA_693 and CGGA_325 glioma patients and divided them into high-risk and low-risk groups with the same cutoff value (3.04). The patients in high-risk group had worse prognosis compared to those in low-risk group (Figures 4G,J). And ROC curve analysis of the signature in CGGA_693 achieved AUCs of 0.719, 0.783, and 0.782 at 1-, 3- and 5-years respectively, indicating a moderately high sensitivity and specificity of the signature in predicting patients’ survival (Figure 4H). Similar result was observed in ROC curve analysis of the signature based on the CGGA_325 (Figure 4K). Further, we revealed the distribution patterns of risk scores and survival status, and found that the survival time of patients with high-risk scores was markedly lower than that of patients with low-risk scores (Figures 4I, 4L).

### Assessment of the Independence of the Prognostic Signature

**FIGURE 5:**
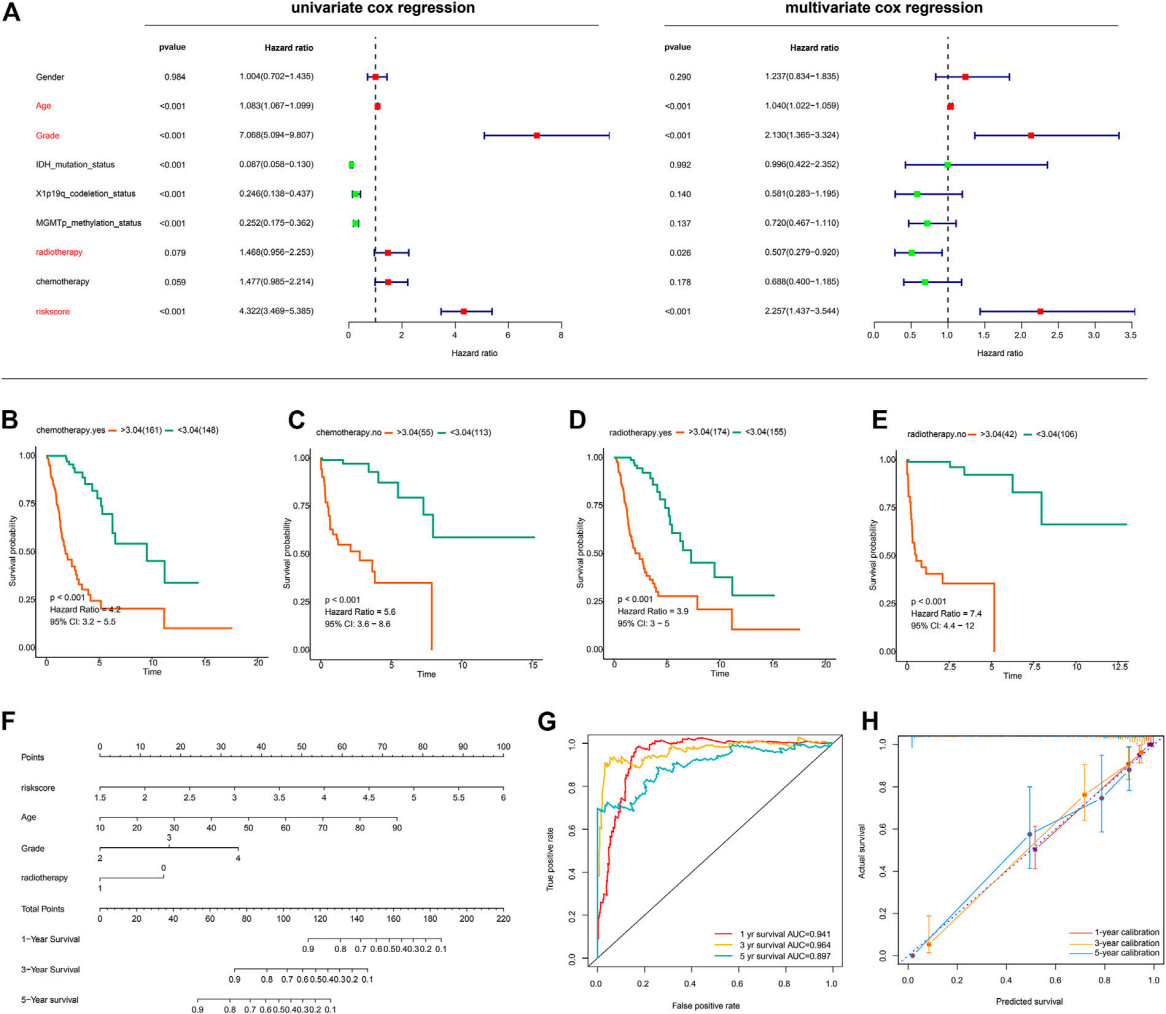
Evaluation of the prognostic signature and establishment of a nomogram **(A)** Univariate and Multivariate Cox regression analysis of the ferroptosis-related lncRNA signature and other clinicopathological features in gliomas **(B–E)** Subgroup survival analysis using groups with chemotherapy **(B)**, without chemotherapy **(C)**, with radiotherapy **(D)**, without radiotherapy **(E) (F)** The nomogram incorporating independent prognostic factors **(G)** ROC curves for the nomogram-based classification at multiple timepoints **(H)** Calibration plots for 1-, 3-, and 5-years survival predictions.

We next sought to determine whether the risk score based on the lncRNA signature is an independent prognostic indicator of OS in glioma patients. The risk score and several clinicopathological variables such as gender, age, grade, IDH mutation status, 1p19q-codeletion status, MGMT promoter methylation status, radiotherapy and chemotherapy were included in univariate and multivariate Cox regression analyses. Univariate Cox regression analysis showed that risk score, age, grade, radiotherapy, IDH mutation status, 1p19q-codeletion status and MGMT promoter methylation status were significantly correlated with the OS of glioma patients, whereas gender and chemotherapy did not show any correlation (Figure 5A, left panel). Four variables were further identified as independent prognostic factors of OS by multivariate analysis, including risk score (HR = 2.257, 95% CI = 1.437–3.544, *p* < 0.001), age (HR = 1.040, 95% CI = 1.022–1.059, *p* < 0.001), grade (HR = 2.130, 95% CI = 1.365–3.324, *p* < 0.001) and radiotherapy (HR = 0.507, 95% CI = 0.279–0.920, *p* = 0.026) (Figure 5A, right panel). Further, subgroup survival analysis showed the signature predicts overall survival in groups with chemotherapy (Figure 5B), without chemotherapy (Figure 5C), with radiotherapy (Figure 5D), and without radiotherapy (Figure 5E).

With the aforementioned independent prognostic factors, we established a nomogram for predicting individual OS. As presented in Figure 5F, the clinicians can predict survival outcome for individuals with known age, tumor grade, and risk score in an intuitive manner. The classification accuracy was 0.941, 0.964, and 0.897 for 1-, 3-, and 5-years survival prediction (Figure 5G). Further, the prediction accuracy was also evaluated with calibration plots, which showed that the nomogram-based prediction was highly consistent with the actual survival at 1-, 3-, and 5-years post-operation (Figure 5H).

### Clinical Correlations of the Prognostic Classifier

**FIGURE 6:**
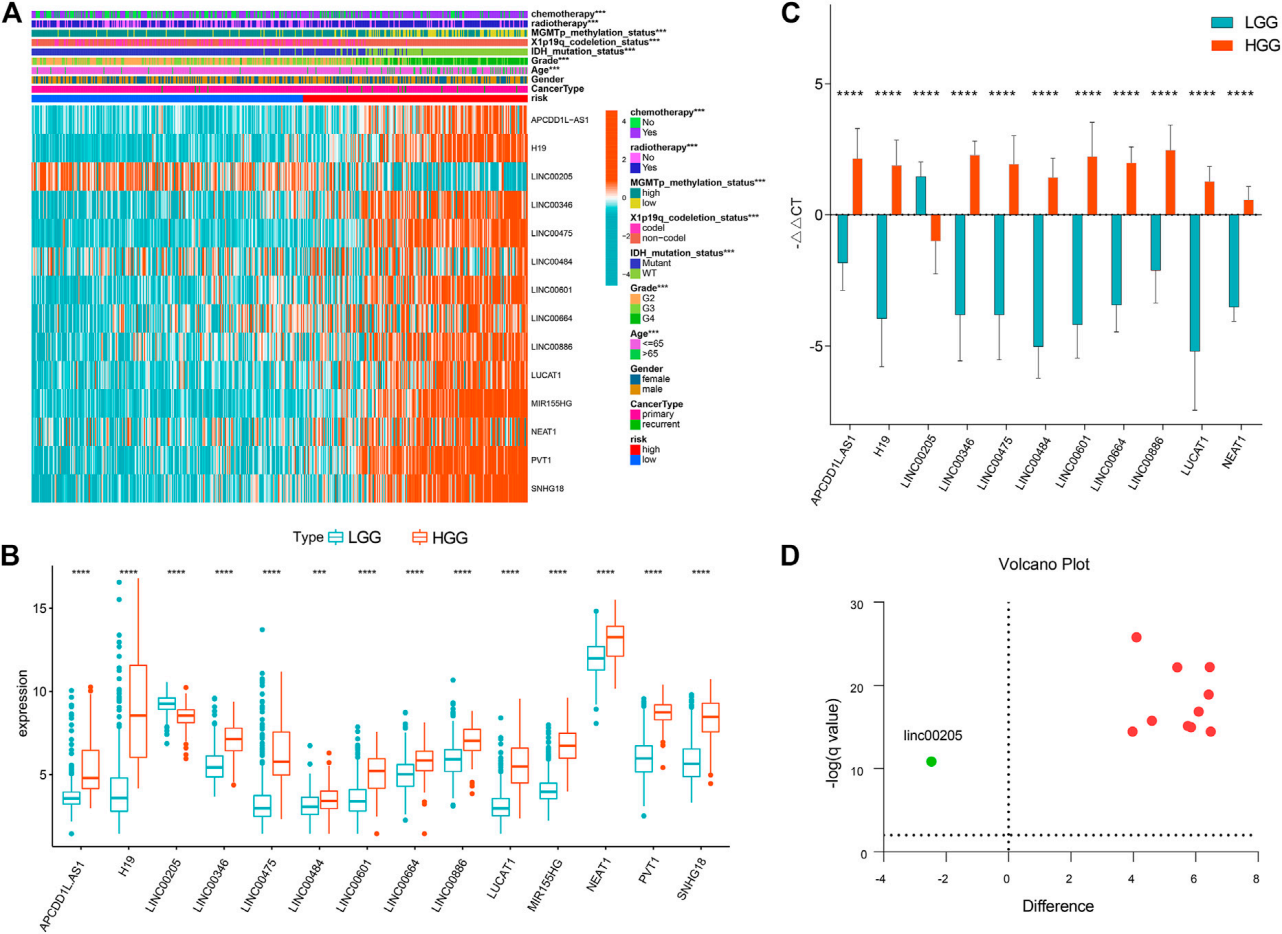
Clinical correlation of the prognostic signature **(A)** Heatmap presenting correlations between the signature and clinical features **(B)** Boxplot showing lncRNA expressions between high-grade and lower-grade samples. ***p < 0.001, ****p < 0.0001 **(C)** Validation for the lncRNA signature in glioma samples using qRT-PCR. ****q < 0.0001. **(D)** Valcano plot showing expressions of the 14 lncRNAs across LGG and HGG. Red dots represent upregulated lncRNAs in HGG and the green dot represents downregulated lncRNA.

As shown in Figure 6A, the prognostic classifier was significantly correlated with multiple clinical features, including age, grade, chemotherapy, radiotherapy and MGMT promoter methylation status, 1p19q-codeletion status, and IDH mutation status (*p* < 0.001). Notably, the correlation between the lncRNA signature and grade was prominent. Further, we analyzed the expressions of 14 lncRNA signatures between high-grade and lower-grade samples, and the between-group results were distinct (*p* < 0.001) (Figure 6B).

### Validations for Long Non-Coding RNAs Expressions With Quantitative Real-Time PCR

Out of the 14 lncRNAs, high expressions of PVT1 and SNHG18 in glioblastoma samples as opposed to LGG was validated in the previous study (Zheng et al., 2021). Likewise, lncRNA MIR155HG was reported in high-grade glioma as compared to LGG (He et al., 2021). As such, we hereby validated the other 11 lncRNAs in clinical specimens. As shown in Figure 6C, we successfully validated 11 lncRNAs, i.e., APCDD1L-AS1, H19, LINC00205, LINC00346, LINC00475, LINC00484, LINC00601, LINC00664, LINC00886, LUCAT1, NEAT1 (q < 0.0001). Apart from LINC00205, all the other lncRNAs were upregulated in HGG (Figures 6C,D).

### Immune Infiltration in the High-Risk Group

**FIGURE 7:**
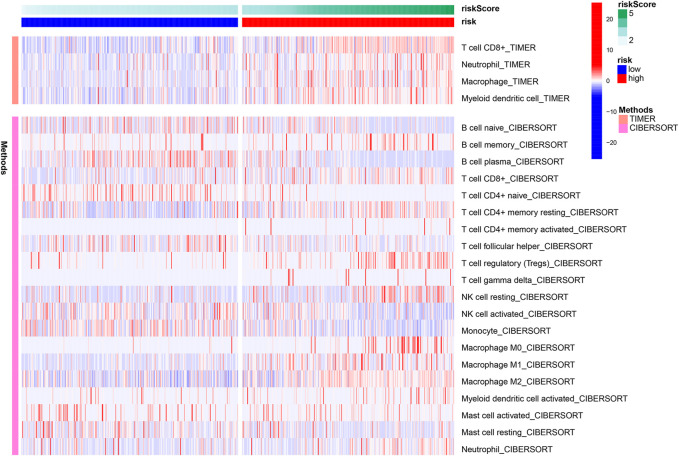
Heatmap presenting immune infiltration correlated to the lncRNA signature.

Using the TIMER algorithm, we identified four types of immune cells upregulated in the high-risk group, i.e., CD8^+^ T cell, neutrophil, macrophage, and myeloid dendritic cell (Figure 7). With CIBERSORT algorithm, more extensive immune cell components were identified, such as B cell memory, T cell CD4^+^ memory (resting and activated), macrophage (M0, M1, and M2), and mast cell activated. These results indicate there was increased immune infiltration in the high-risk group defined by the ferroptosis-related lncRNA signature (Figure 7).

## Discussion

Due to the inherent heterogeneity of glioma, the current WHO classification of gliomas does not sufficiently characterize the distinct survival outcomes. Even with well-established molecular markers, the prognosis stratification and treatment of glioma patients remain challenges. Ferroptosis is involved in the development and progression of cancers and its pivotal role in glioma has been recently unveiled (Liu et al., 2020). In this context, the present study aimed to establish a novel ferroptosis-related lncRNA signature for glioma.

Our study identified 30 ferroptosis-related lncRNAs in association with tumor grade and microenvironment via WGCNA. With the 30 hub lncRNAs, we developed a lncRNA signature for prognosis prediction. The ferroptosis-related lncRNA signature was clinically relevant with distinct stratification of survival chance in patients with glioma in two independent cohorts. Robust predictive accuracies of the signature were present across different datasets at multiple timepoints. With univariate and multivariate regressions, we demonstrated that the risk score based on the lncRNA signature is an independent prognostic indicator after clinical factors were adjusted. The nomogram incorporating age, tumor grade, radiotherapy and risk score was observed to be accurate in both classification and prediction performance. What’s more, the signature-based stratification was observed to be correlated with extensive clinical characteristics, including tumor grade, MGMT promoter methylation status, 1p19q-codeletion status, and IDH mutation status. Further, increased immune infiltration was observed in the high-risk group as defined by the signature, which contributed to detrimental survival outcomes.

The ferroptosis-related lncRNA signature highlighted a total of 14 lncRNAs, i.e., APCDD1L-AS1, H19, LINC00205, LINC00346, LINC00475, LINC00484, LINC00601, LINC00664, LINC00886, LUCAT1, MIR155HG, NEAT1, PVT1, and SNHG18. Out of the 14 lncRNAs, eight of them were previously reported. H19 was reported to be upregulated in high-grade glioma and facilitated the proliferation of diffuse intrinsic pontine glioma (Roig-Carles et al., 2021). LINC00346 promotes cell migration, proliferation, and apoptosis by targeting ROCK1(Chen et al., 2020) and miR-128-3p/SZRD1 axis (Geng et al., 2020) in glioma. Overexpression of LINC00475 was observed in hypoxic gliomas and silencing LINC00475 results in suppression of tumor proliferation, migration, as well as invasion (Yu et al., 2020a). By regulating the miR-141-3p/YAP1 axis, LINC00475 facilitates tumor progression of glioma (Yu et al., 2020b). LUCAT1 is an oncogenic molecule in glioma, and its knockdown induced inhibition of cell viability and invasion by regulating miR-375 in glioma (Gao et al., 2018). LncRNA miR155HG contributes to tumor growth and progression in glioblastoma (Wu et al., 2019), and its prognostic value was also identified in previous bioinformatic analysis (Zheng et al., 2021). NEAT1 demonstrated to be a contributor to glioma cell migration, invasion, and tumor progression (Chen et al., 2018; Zhou et al., 2018). PVT1 promotes tumorigenesis and cancer progression of glioma via regulation of miR-128-3p/GREM1 Axis and BMP signaling pathway (Fu et al., 2018). Upregulation of SNHG18 promotes resistance to radiotherapy in glioma by repressing Semaphorin 5A (Zheng et al., 2016). High expressions of PVT1 and SNHG18 in glioblastoma samples as opposed to LGG was validated in the previous study (Zheng et al., 2021). Notably, the role of lncRNAs APCDD1L-AS1, LINC00205, LINC00484, LINC00601, LINC00664, and LINC00886 was first reported and validated in the present study.

Zheng et al. (Zheng et al., 2021) established a prognostic ferroptosis-Related lncRNAs signature correlated with immune landscape and radiotherapy response in Glioma. However, they applied median risk scores for three cohorts with a half-by-half classification in each cohort, thereby leading to potential false-positive results. Notably, there was no other report of ferroptosis-related lncRNA signature in gliomas. By contrast, the present study reports a ferroptosis-related lncRNA signature, which exhibits a distinct separation of survival rates in glioma patients with the same cutoff value across different cohorts. Further, expression levels of the ferroptosis-related lncRNAs signature were also validated between high-grade and lower-grade samples using qPCR. It could be translated to clinical settings as a test panel for informing individualized prognosis. Therapies targeting these lncRNAs could hold promise for enhanced efficacy. However, a lack of functional validation is considered as a major limitation in the present study. The functional implications of the lncRNAs require further validation with empirical data.

## Conclusion

The present study established a ferroptosis-related lncRNA signature that could effectively stratify the prognosis of glioma patients, with superior predictive performance to all clinical index. These findings may pave the way for developing novel biomarkers for prognosis and treatments in gliomas.

## Data Availability

The datasets presented in this study can be found in online repositories. The names of the repository/repositories and accession number(s) can be found in the article.
